# Revisiting partition priming in judgement under uncertainty: replication and extension Registered Report of Fox and Rottenstreich (2003)

**DOI:** 10.1098/rsos.250669

**Published:** 2025-07-09

**Authors:** Kerou Ding, Gilad Feldman

**Affiliations:** ^1^ Department of Psychology, The University of Hong Kong, Hong Kong, Hong Kong

**Keywords:** bias, judgement and decision making, partition dependence, ignorance prior, risk, registered report, replication

## Abstract

Partition dependence is the phenomenon in which individuals’ evaluations of probabilities are influenced by the partitioning of the information in the way the information is presented or framed. In a Registered Report experiment with an American online Prolific sample (*N* = 603), we conducted a replication and extension of Studies 1a, 1b, 3 and 4 from a classic article by Fox and Rottenstreich (Fox & Rottenstreich 2003 *Psychol. Sci*. **14**, 195–200 (doi:10.1111/1467-9280.02431)) demonstrating the phenomenon. They showed that participants’ estimations of an event’s likelihood shifted based on minor adjustments of the framing that suggested a change in partitioning perspective (‘ignorance prior’ priming). Based on a pre-registered replication evaluation criterion, we concluded a mostly successful replication of the findings of Fox and Rottenstreich (2003). Specifically, we found support for partition dependence in scenarios from Study 1a Items 1 and 2, Study 1b and Study 3, with weaker effects, yet with no consistent support for Study 1a Item 3 and Study 4. Our extensions further explored the robustness of partition dependence by examining participants’ probability evaluations of complementary events (happen versus not happen) and the impact of task order on judgement and decision making. Overall, the findings suggest support for partition dependence, yet as more nuanced and context-dependent than expected, and the need for further research to understand its mechanisms, robustness and implications across different contexts. Materials, data and code are available on at https://osf.io/g9czs/. This Registered Report has been officially endorsed by Peer Community in Registered Reports: https://doi.org/10.24072/pci.rr.100877.

## 1. Background

When lacking specific knowledge about a situation, people tend to rely on an ‘ignorance prior’, an assumption that assigns equal chances to different possibilities. Yet, the perceived ignorance prior may depend on the way the information is presented, and how the person mentally maps the available possibilities. Fox & Rottenstreich [1] demonstrated that a slight change in the framing of the same problem, partitioning, may lead to the use of different ignorance priors and therefore shift evaluations of perceptions of the probabilities. For example, when asked about the weather the following week with no relevant information, the questions ‘will Sunday be the hottest day next week?’ and ‘will Sunday be hotter than all other days next week?’ might trigger different ignorance priors. The first might trigger a 1/2 versus 1/2 given a mental split of ‘hotter than rest of the week’ versus ‘not hotter’, whereas the latter might trigger a 1/7 versus 6/7 given a mental comparison of Sunday against each of the other 6 days.

We conducted an independent close replication and extension Registered Report of Fox & Rottenstreich [1], as one of the classic articles of the partition dependence phenomenon. We begin by introducing the literature on ignorance priors and the chosen article for replication [1]. We then discuss our motivations for the current replication, review their work and outline the chosen studies from the target article, their experimental design and our adaptations and extensions.

### 1.1. Ignorance priors and partition dependence

Decision making and risk analysis often involve the assessment of probabilities for uncertain events. Individuals rely on the perceived number of potential events in making likelihood estimations when no other information is provided [1–3]. For example, if there are 10 candidates in a competition and no information is available regarding their abilities, one might assign a probability of 1/10 to each candidate’s chance of winning the competition. In this case, 1/10 is referred to as the ‘ignorance prior’ of each candidate winning the competition. An ignorance prior denotes a default judgement that assigns equal probabilities to all potential possibilities in a given query, in the absence of any other relevant information.

While the reliance on ignorance priors may initially seem intuitively appealing, it may be sensitive to presentation and framing [1,3]. To illustrate, consider the competition example previously discussed. Imagine an assessor, lacking detailed information on the competition’s difficulty and scoring system, is tasked with judging the likelihood that the competition winner receives a score lower than 50. In this scenario, the question tends to elicit a binary partition: {score lower than 50; score higher than 50}. However, if the task is framed so that the task is to estimate the likelihood that the winner’s score falls between 50 and 100, then the partition tends to elicit a threefold partition: {score lower than 50; score between 50 and 100; score higher than 100}. Consequently, this partitioning increases the likelihood that each of those three possibilities is assigned an ignorance prior of 1/3. This adjustment in how the stated space is partitioned might shift the assessed probability [1,3,4]. In other words, the default ignorance prior assigned by an assessor is partition dependent.

The influence of partition dependence on decision making has been observed across different contexts. Fox *et al.* [5] reported that the subjective grouping of options influences decision making and resource allocation, recently successfully replicated by Li & Feldman [6]. For example, in a study where participants were asked to select candies from three bowls containing a total of four types of candy, they tended to spread their choices not only across the bowls but also across the different types of candy. This suggested that the distribution of choices was influenced by the way the candies were subjectively grouped or partitioned into different bowls by the experimenters. Feng *et al.* [7] showed that when job applicants were partitioned on the basis of various criteria such as gender, nationality and university, individuals tended to select a more diverse pool of candidates within each specific partitioned criterion. This effect was observed even among experts (seasoned human resource professionals).

The assumption of the ‘principle of insufficient reason’ is the absence of any relevant information favouring one outcome over another, and yet according to Fox & Rottenstreich [1] individuals often possess some knowledge they consider to be relevant in many real-life situations. In such cases, people may resort to a combination of evaluative strategies and the ‘principle of insufficient reason’. Thus, individuals’ judged probabilities may exhibit a consistent bias towards partition dependence under conditions of both ignorance (complete absence of relevant knowledge) and uncertainty (possession of relevant knowledge to some extent).

### 1.2. Choice of study for replication: Fox and Rottenstreich (2003)

We embarked on a replication and extension Registered Report of Fox & Rottenstreich [1]. We aimed to revisit the phenomenon to examine the reproducibility and replicability of the findings with an independent well-powered replication and extension Registered Report. This follows the recent growing recognition of the importance of reproducibility and replicability in psychological science [8,9].

We chose Fox & Rottenstreich [1] based on several factors: its academic and practical impact, the potential for improvements in methodology and the mixed findings in the literature.

Fox & Rottenstreich’s [1] findings have had an impact on scholarly research in the domains of judgement bias, subjective probability estimation and risk assessment. At the time of writing (February 2025), there were 255 Google Scholar citations of the article and many important follow-up theoretical and empirical articles, such as Fox & Clemen [3] and See *et al.*’s [4] research on partition-dependent ignorance priors, exploring how these priors were influenced by individuals’ expertise and confidence levels.

There is some indication for mixed findings in the partition dependence literature. For example, Reichelson *et al.* [10] attempted to replicate the candy bowl study previously conducted by Fox *et al.* [5], recruiting both children and adults. They concluded no evidence supporting the impact of candy partition on participants’ choices, inconsistent with the findings of Fox *et al.* [5]. Their findings were in contrast to our successful replication of the same article as a Registered Report [6].

We attempted to analyse Fox & Rottenstreich’s [1] studies. The methods and the statistical tests varied across the different studies, despite having very similar designs and hypotheses, with inconsistent reporting of key details for both the procedures and the results. Their brief reporting proved a challenge in deducing the procedure and materials for a replication and comparing the different scenarios. We believe that revisiting classics like our target article with reproductions and replications helps clarify important needed details and increases the quality of the literature building on these findings.

### 1.3. Selection of studies to replicate: Studies 1a, 1b, 3 and 4

Fox & Rottenstreich [1] investigated ignorance priors and partition dependence in five studies (Studies 1a, 1b, 2, 3 and 4). Studies 1a and 1b focused on judgement under ignorance or near-ignorance situations, where implementing evaluative approaches is difficult, thereby highlighting reliance on the ignorance prior. In Study 1a, questions presented in the binary (1/2) case-prime condition elicited higher ignorance priors compared to the *N*-fold (1/*n*) class-prime condition. To address an alternative interpretation suggesting that class formulations might depress probability judgements relative to case formulations, Study 1b involved a situation in which there were higher expected class-primed ignorance priors than case-primed ignorance priors.

Studies 2 and 3 investigated partition dependence in judgement under uncertainty, where individuals can draw on relevant knowledge and may rely on both evaluative assessment and the ignorance prior. Study 2 tasked participants with estimating their score range within a group, leveraging their knowledge of other group members. In Study 3, participants estimated the probability of a candidate’s success in a job application after reading a recommendation letter excerpt of that candidate.

While Studies 1a, 1b, 2 and 3 centred on judgements, Study 4 explored partition dependence in decision making. Participants were presented with two options and were informed that some would receive a real reward in accordance with their choices. The first option did not involve probability estimation and remained constant across the two conditions. The second option was riskier than the first option. It was formulated with either a case-primed or a class-primed ignorance prior in either the case or the class condition (table 7). Participants’ tendency to choose the second option implicitly indicated their probability estimation of the event described by the second option.

We focused our investigation on the studies examining how partition dependence affects judgements and decision making under ignorance and uncertainty: Studies 1a, 1b, 3 and 4 (table 1). We chose not to replicate Study 2 given that it was conducted in an in-class setting with almost no information provided about methods, procedure and results.

**Table 1.** Studies 1a, 1b, 3 and 4: comparison of design*.*

study	1a	1b	3	4
situation	ignorance	ignorance	uncertainty	ignorance
task	probability judgement	probability judgement	probability judgement	decision making
dependent variable	numerical probability	numerical probability	numerical probability	categorical decision outcome

In situations of ignorance, participants have little or no relevant knowledge. In situations of uncertainty, participants have the opportunity to apply relevant knowledge to some extent.

### 1.4. Fox and Rottenstreich (2003): hypotheses and findings

We did our best to analyse the brief details provided by Fox & Rottenstreich [1] and summarize our interpretation of the different designs in table 1, their hypotheses and findings in table 2, and their descriptives and statistical results in table 3.

**Table 2.** Studies 1a, 1b, 3 and 4: summary of hypotheses and findings.

study	scenario	hypothesis	findings of the target article
1a	judgements under ignorance	individuals utilize partition-dependent probabilities when forming judgements under ignorance when the case prime facilitates a greater ignorance prior of an event’s occurrence than the class prime (1/2 > 1/*n*)	participants showed a bias towards partition-dependent ignorance priors. Specifically, participants’ judgements differed between the case-prime and the class-prime condition: (1) responses were higher in the case-prime condition with an ignorance prior of 1/2 than in the class-prime condition with an ignorance prior of 1/*n*, indicated by both means and medians of judged probabilities (2) 1/2 responses were more common under the case prime than under the class prime. 1/*n* responses were more common under the class prime than under the case prime
1b	judgements under ignorance	individuals utilize partition-dependent probabilities when forming judgements under ignorance when the class prime facilitates a higher default probability than the case prime (1/2 < *x*/*n*)	participants showed a bias towards partition-dependent ignorance priors. Specifically, participants’ judgements differed between the case-prime and the class-prime condition: (1) responses were lower in the case-prime condition with an ignorance prior of 1/2 than in the class-prime condition with an ignorance prior of *x*/*n*, indicating by both means and medians of judged probabilities (2) *x*/*n* responses were more common under the class prime than under the case prime
3	judgements under uncertainty	individuals utilize partition-dependent probabilities when forming judgements under uncertainty (1/2 > 1/*n*)	participants showed a bias towards partition-dependent ignorance priors. Specifically, participants’ judgements differed between the case-prime and the class-prime condition: (1) responses were higher in the case-prime condition with an ignorance prior of 1/2 than in the class-prime condition with an ignorance prior of 1/*n*, indicated by comparison of medians of judged probabilities (2) 1/*n* responses were more common under the class prime than under the case prime
4	decisions under ignorance	individuals utilize partition-dependent probabilities when making decisions under ignorance (1/2 > 1/*n*)	participants showed a bias towards partition-dependent ignorance priors. Specifically, under the case prime, the risker choice had an ignorance prior of 1/2, while under the class prime, the risker choice had an ignorance prior of 1/*n*. A larger proportion of participants in the case-prime condition made decisions favouring the risker choice when compared to those in the class-prime condition, 23% versus 11%, *z* = 1.95, *p* < 0.05, one-tailed

Under case prime, the ignorance prior of the target event’s occurrence is 1/2. In Studies 1a, 3 and 4, under class prime, the ignorance prior of the target event’s occurrence is 1/*n* where *n* stands for the number of all possible events in the question. In Study 1b, participants were tasked with estimating the probability of a collection of single events, with *x* denoting the number of events in that collection. In Study 1a, the comparison between the proportions of 1/2 and 1/*n* responses relied solely on proportion figures. No statistical test results were provided for this comparison. In Studies 1b, 3 and 4, the *χ*^2^ test was used to compare the proportions of 1/*n* or *x*/*n* judgements between the class-prime and case-prime conditions.

**Table 3.** Studies 1a, 1b, 3 and 4: summary of reported results.

		study 1a	study 1b	study 3	study 4
dependent variable		probability estimation	probability estimation	probability estimation	choice
*n*	case	41	22	32	74
	class	53	20	41	70
*Med*	case	—	0.69	0.40	
	class	—	0.71	0.23	
*M*	case	—	0.61	—	
	class	—	0.72	—	
proportion of case-primed 1/2 responses	case	19%	—	—	
	class	8%	—	—	taking the bet:
proportion of class-primed 1/*n* (*x*/*n*) responses	case	19%	23%	6%	23%
	class	49%	55%	28%	11%
Mann–Whitney *U*	*U*-statistic	—	—	—	
	*p*	<0.01	<0.05	<0.05	
Welch’s *t*	*t*	—	2.75	—	
	d.f.	—	22	—	
	*p*	—	= 0.01	—	
	*d*	0.55 [0.13, 0.96]	−0.87 [−1.51, −0.22]	0.47 [0.00, 0.93]	
	*N* required	174	71	238	
*χ* ^2^	*χ* ^2^	—	4.63	5.67	
	d.f.	—	1	1	
	*p*	—	<0.05	<0.05	
	*z*				1.95
	*p*				0.05
	*h*	0.65 [0.24, 1.06]	0.67 [0.07, 1.28]	0.62 [0.16, 1.08]	0.32 [0.00, 0.65]
	*N* required	136	121	164	508

*Med*, median; *M*, mean; —, results were not reported in the target article. The range of probability estimation spans from 0 (0%) to 1 (100%). For Studies 1a and 3, Cohen’s *d* was estimated from the *p*-values of the Mann–Whitney *U*-test using the esc package [11], despite these analyses being meant for *t*‐test *p*-values, as we assumed that 0.01 is a good enough proxy for a signal regardless of analysis. For Study 1b, Cohen’s *d* was calculated from Welch’s *t* and using the effectsize package [12]. See accompanying Rmarkdown for effect size calculations. We aligned all effects of Welch’s *t* tests (Cohen’s *d*) to go in the same direction with case condition higher than class condition, yet Study 1b was meant to demonstrate class condition higher than case condition, and so the effects are coded as negative.

In Fox & Rottenstreich’s [1] studies, partition priming was manipulated by the linguistic reformulation of a probability query. In what they referred to as a ‘case partition’, the target event either will or will not occur. For example, the question ‘What is the probability that Sunday will be hotter than every other day next week?’ would facilitate a binary case partition: Sunday either will or will not be hotter than every other day. In what they referred to as a ‘class partition’, the probability of a target event is compared against an entire class of possible events. For example, the question ‘What is the probability that the hottest day of the week will be Sunday?’ would facilitate a sevenfold class partition: {Sunday hottest, Monday hottest, …, Saturday hottest}. Participants were randomly assigned to make probability estimations in either the case-prime condition, where all the probability questions featured case partition, or the class-prime condition, where all the probability questions featured class partition. The only distinction between the two experimental conditions was in its linguistic formulation, the target events remaining consistent in nature across both conditions.

Given the reporting standards at the time, the reporting of both methodology and the findings were brief with many missing details. In Study 1a, three pairs of questions were presented in each condition. They reported a Mann–Whitney *U*-test for all three items and a result of *p* < 0.01. In Studies 2b and 3, they compared the class-prime and case-prime conditions proportions of judgements that were precisely in accordance with the class-prime ignorance prior (1/*n*; *χ*^2^ test).

### 1.5. Extensions: complementary hypotheses

We aimed to extend the replication study by incorporating pairs of complementary hypotheses into Studies 1b and 3. Complementary hypotheses predict mutually exclusive outcomes for the same event. For instance, ‘Sunday will be the hottest next week’ and ‘Sunday will *not* be the hottest next week’ constitute a pair of complementary hypotheses. The target article only included two complementary hypotheses for the three items in Study 1a, but not in Studies 1b and 3. Fox & Rottenstreich [1] did not elucidate the rationale behind adding the alternative hypothesis. Furthermore, it was unclear whether each participant encountered both mutually exclusive hypotheses or if half of the participants in each condition estimated the probability of a target event occurring, while the other half estimated the probability of the positive target *not* occurring. Our best guess based on the description ‘assigned probabilities to three pairs of complementary hypotheses’ is that participants rated both hypotheses.

We therefore decided to expand the original design by incorporating pairs of complementary hypotheses also into Studies 1b and 3. This expansion offers several important advantages. First, it allows for a more comprehensive comparative analysis of the partition priming effects of ‘happen’ and ‘not happen’ responses across the three estimation studies—Studies 1a, 1b and 3. Through this approach, we can explore whether participants demonstrate the same pattern of reliance on ignorance priors and partition dependence when estimating negative complementary events as their mutually exclusive positive events, and for several scenarios. Second, seeing both hypotheses helps increase the likelihood that participants are processing a fuller range of events, and allows us to indirectly test for both attentiveness and understanding, given that the two probabilities should sum to 100 or close (if there are rounding issues). We contemplated whether to force the two estimations to have to add up to 100, yet decided to instead allow participants to enter whatever approximations to allow for the possibility that participants were systematically deviating from our expected use of the complementary hypotheses, which we could then analyse using exploratory analyses.

### 1.6. Pre-registration and open science

We provided all materials, data and code on: https://osf.io/g9czs/. This Registered Report was submitted to *Royal Society Open Science* following peer review and recommendation for Stage 2 acceptance at the Peer Community In (PCI) Registered Reports platform. Full details of the peer review and recommendation of the paper at PCI Registered Reports may be found at the links below. After submission to the journal, the paper received no additional external peer review, but was accepted on the basis of the Editor’s recommendation according to the RSOS PCI Registered Reports policy (https://royalsocietypublishing.org/rsos/registered-reports#PCIRR). Stage 1 recommendation and review history: https://rr.peercommunityin.org/articles/rec?id=670; https://osf.io/px6vb (our frozen pre-registration version of the entire Stage 1 packet: https://osf.io/k4up5). Stage 2 recommendation and review history: Espinosa [13]; https://doi.org/10.24072/pci.rr.100877. All measures, manipulations, exclusions conducted for this investigation are reported and data collection was completed before analyses. The project was part of a large mass replications and extensions project, which received ethics approval from the University of Hong Kong (no. EA220438). This Registered Report was written based on the Registered Report template by Feldman [14].

## 2. Method

### 2.1. Power and sensitivity analyses

We first calculated effect sizes and conducted a power analysis based on the effects reported in the target article. We used information from the target article (summarized in table 3) to calculate effect sizes and confidence intervals using R v. 4.3.3 [15] with the help of a guide by Jané *et al.* [16]. We used the effectsize package [12], the esc package [11] and pwrss [17] to compute Cohen’s *d* and Cohen’s *h*.

We concluded that the minimum required sample size was 508 participants in total. We provide more information regarding these calculations in the ‘Power analysis of the original study effect to assess the required sample for replication’ subsection of the electronic supplementary material.

Moreover, given the likelihood that the target article’s effects were an overestimation and the difficulty in computing the effect sizes of Studies 1a, 1b and 3, we used the ‘small-telescope’ approach [18] aiming for enough power to detect effects much weaker than those reported by the original study (*d*_33%_) with the general rule of thumb to multiply the largest sample in the target by 2.5. This resulted in a sample of 360 (2.5 times the sample size of Study 4, 144). Given that 360 is less than 508, we maintained the sample size at 508. Accounting for our integrated design, and allowing for the potential of additional analyses, we aimed for a larger total sample of 600 participants, more than four times larger than any of the samples in the target article.

We conducted a sensitivity analysis using Gpower [19] which indicated that a sample of 600 would allow the detection of effect sizes of Cohen’s *d =* 0.27 for independent *t*-tests in Studies 1a, 1b and 3, and Cohen’s *w* = 0.14 for chi-square to compare proportions reported in Studies 1a, 1b, 3 and 4 (all alpha = 5%, power = 95%, one-tail). These results correspond to weak to medium effects in social psychology [16]. We also note that we completed a similar well-powered Replication Registered Report project conducted with Peer Community in Registered Reports (PCIRR) on diversification bias and partition dependence [6], with very large effects overall but with one small effect of *d* = 0.27, which the target sample was sufficiently powered to detect.

### 2.2. Participants

We recruited a total of 603 US American participants from Prolific [20] (*M_age_* = 42.96, s.d. = 13.96; 333 females, 261 males, 9 other or did not disclose). We note that 707 subjects began the survey but 104 did not proceed beyond the consent and verifications. We summarize a comparison of the target article sample and the replication samples in table 4.

**Table 4.** Differences and similarities between original study and replication.

	Fox & Rottenstreich [1]	US Americans on Prolific
sample size	Study 1a: 94 Study 1b: 42 Study 3: 73 Study 4: 144 overall: 353	603
geographic origin	US American	US American
gender	not reported	333 males, 261 females, 9 other/did not disclose
median age (years)	not reported	40
average age (years)	not reported	42.96
standard deviation age (years)	not reported	13.96
age range (years)	not reported	19−95
medium (location)	not reported (potentially in person on a US university campus)	computer (online)
compensation	Studies 1a and 3: 1 dollar reward Study 1b: a donation to charity (the value was not mentioned) Study 4: random chance of receiving rewards in line with the participant’s choice	30 participants taking part in the pretest received £0.95 (~$1.20) as compensation. Each of the remaining participants received £1.05 (~$1.33). The payment was determined based on the estimated completion time from the pretest, aiming for £9 (~$11.37) per hour
year	2003 or earlier	2024

It is unlikely that the maximum age range in the replication was indeed 95, it is more likely that some participants were misreporting, yet we report the demographics as is. We reported compensation in British pounds given that Prolific charges researchers in pounds. US dollar estimates are according to the exchange rate in February 2025 (1 pound = 1.2635 US dollars).

We targeted US Americans using Prolific’s filters. We prescreened our participants using the following standards: ‘Nationality: United States’, ‘Country of birth: United States’, ‘Place of most time spent before turning 18: United States’, ‘Minimum Approval Rate: 95, Maximum Approval Rate: 100’, ‘Minimum Submissions: 100, Maximum Submissions: 10 000’.

We first pretested the survey duration and technical feedback with 30 participants to make sure our time run estimate was accurate and adjusted pay as needed. The data of the 30 pretest participants were not analysed other than to assess survey completion duration, feedback regarding possible technical issues and payment and needed pay adjustments. We pre-registered that unless there were serious technical issues that affect data quality and require survey modification, these participants will be included in the overall analysis. We did not identify technical issues with the pretest.

### 2.3. Design: replication and extension

In the target article, Studies 1a, 1b, 3 and 4 were conducted separately with independent samples. We ran the four studies together in a single unified data collection, with all scenarios from the four studies presented in random order. Participants were first randomly allocated to either the case-prime or the class-prime condition. This unified design combining replications of several studies into a singular data collection was previously tested successfully in many of the replications and extensions conducted by our team [21–23], also with one successful replication Registered Report on partition dependence with no impact of order effects [6]. We believe that this design is especially powerful in addressing concerns about the target sample (e.g. naivety and attentiveness) when some studies replicate successfully whereas others do not, as has happened in this replication, as well as in allowing for drawing inferences about links between the different studies and consistency in participants’ responding to similar decision-making paradigms. We return to this point in our discussion.

We summarize the experimental designs in tables 5 and 6, and our adjustments to the target article in table 7. The baseline main effect design mirroring the target article was a simple between-subjects two-partition contrast of case prime versus class prime. The fuller design including the within-subject extensions for Studies 1a/b and 3 were a 2 (case prime versus class prime; between) by 2 (‘happen’ versus ‘not happen’; within) by 5 (all scenarios; within) mixed design. The presentation order of task items (happen versus not happen) was also randomized.

**Table 5.** Studies 1a, 1b and 3: replication and extension experimental design [between]*.*

case-prime condition	class-prime condition
binary case partition: {a target event will happen versus a target event will not happen}	*N*-fold class partition {event 1 will happen, event 2 will happen, … event *n* will happen}
ignorance prior: 1/2	*n* = number of comparable events in a query. Ignorance prior: 1/*n*
DV: probability judgements [replication] Participants make probability judgements of a target event which includes a pair of complementary hypotheses There are three items in Study 1a: Item 1 target event: the noontime temperature at O’Hare airport on sunday will be higher than other days next week Item 2 target event: the University of Michigan will win the scoring title of the Big Ten Conference in Women’s Iacrosse for the upcoming season Item 3 target event: International Business Machines Corporation’s (IBM) stock price will rise by more than any other stock on the Dow Jones Industrial Average (DJIA) tomorrow There is one item in Studies 1b and 3, respectively Study 1b target event: the warmest day of the week next week (afternoon high temperature) will fall on a weekday (Mon–Fri) rather than the weekend (Sat–Sun) Study 3 target event: K.T. will be offered a job this year by ACME Happen: estimation of the probability that a target event will happen: Scale: 0 = *target event will not happen*; 1 = *target event will happen* Not happen: estimation of the probability that a target event will not happen: Scale: 0 = *target event will happen*; 1 = *target event will not happen*	

An ignorance prior is a default judgement which assigns equal likelihood to each comparable event in a query when the judge has little or no relevant knowledge of the events. Study 1a explores judgements under conditions of ignorance, where participants have limited or no access to relevant knowledge. Study 1b investigated judgements under conditions of ignorance, whereas Study 3 explored judgements under uncertainty. In situations of uncertainty, participants have the opportunity to apply relevant knowledge and may employ a combination of evaluative strategies and ignorance priors. In Study 3, participants are informed that ACME Corporation plans to extend job offers to 10 out of 100 applicants. Subsequently, they read excerpts from a recommendation letter portraying applicant K.T. as cheerful, bright and hardworking, but somewhat set in her ways.

**Table 6.** Study 4: replication and extension experimental design [between].

case-prime condition	class-prime condition
option 1: receive $10 for sure	option 1: receive $10 for sure
option 2: receive $50 if IBM’s price per share rises by a greater percentage today than any other stock on the DJIA	option 2: receive $50 if the stock whose price per share rises by the greatest percentage on the DJIA today is IBM
ignorance prior of option 2: 1/2	ignorance prior of option 2: 1/30
DV: decision [replication] participants make decisions between two options scale (categorical): option 1; option 2	

Study 4 examined decisions under ignorance. All participants were reminded that ‘the Dow-Jones Industrial Average (DJIA) consists of 30 large industrial stocks, including International Business Machines Corporation (IBM)’.

**Table 7.** Replication and extension adjustments to the target article’s methods and design.

study	factor	target article	adjustment in current study	reason for change/justifications
1a	Item 2 (see table 6 for details)	the target event was the University of Illinois winning the Big Ten Conference in Women’s Iacrosse	we changed the target event to the University of Michigan winning the Big Ten Conference in Women’s Iacrosse	the University of Illinois is not currently competing in the Big Ten Conference in Women’s Iacrosse
1a	Item 2 ignorance prior and information presentation	1/10	1/7 explicitly informing participants regarding the number of teams (7)	Fox & Rottenstreich [1] did not mention to the participants that the Big Ten Conference comprised 11 teams. Given the relative obscurity of the league and its suggestive name, they assumed that most participants would perceive the league as having 10 teams
1a	Item 3 (see table 5 for details)	the target event was GM’s stock price rising by the greatest amount on the DJIA	we changed the target event into IBM’s stock price rising by the greatest amount on the DJIA	GM is not a component of the DJIA currently (2024)
1b, 3	extra items	only Study 1a incorporated pairs of complementary hypotheses of the same event	we added a complementary hypothesis to each item in Studies 1b and 3	refer to the ‘extensions’ section for details
1a, 1b, 3	data format: probability	participants recorded probability estimations in both fractions and decimals	we specifically asked our participants to record probability estimation in percentages, fraction answers were not allowed. We clarified in the instructions that participants could convert a fraction response into a percentage	consistency, ensuring the same processing mode across participants, allowing for answer validation in qualtrics, and for more accurate data analysis that does not require conversions
1a, 1b, 3	classification of responses aligned with ignorance priors	±1% or exact values of the ignorance prior	±5% of the ignorance prior	consistency across Studies 1a, 1b and 3
1a	data analysis	Mann–Whitney *U*-test	Mann–Whitney *U*-test, Welch’s *t*‐test and chi-squared test (the proportion of case-primed responses and class-primed responses in the two conditions)	robustness checks and improving on the target’s data analysis
1b	data analysis	Mann–Whitney *U*-test, Welch’s *t*‐test and chi-squared test (the proportion of class-primed responses in the two conditions)	Mann–Whitney *U*-test, Welch’s *t*‐test and chi-squared test (the proportion of case-primed responses and class-primed responses in the two conditions)	robustness checks and improving on the target’s data analysis
3	data analysis	chi-squared test (the proportion of class-primed responses in the two conditions)	Mann–Whitney *U* test Welch’s *t*‐test and chi-squared test (the proportion of case-primed responses and class-primed responses in the two conditions)	robustness checks and refining data analysis strategy
1a (Item 3) and 4	data analysis	there was no analysis for order effect because Studies 1a and 4 were conducted independently with different participants	we will test the order effect. Specifically, we will examine whether the order of task presentation (Study 1a Item 3 and Study 4) influences decision-making outcomes in Study 4	to explore whether the reliance on ignorance priors is influenced by the use of a numerical response scale or not
1a, 1b and 3	data analysis	Mann–Whitney *U*-test, Welch’s *t*‐test and chi-squared test	an additional 2 (condition: between-subjects) × 5 (question: within-subjects) × 2 (event: within-subjects) mixed ANOVA	a mixed ANOVA allows for the examination of interaction effects between condition (between-subjects) and question and event (within-subjects), providing a comprehensive analysis of how these factors influence participants’ responses
1a, 1b, 3 and 4	compensation	Studies 1a and 3: 1 dollar reward Study 1b: a donation to charity (the value was not mentioned) Study 4: random chance of receiving rewards in line with the participant’s choice	nominal prolific pay for a single data collection of all four studies	the original studies exclusively targeted university students, affording Fox & Rottenstreich [1] greater flexibility in compensation adjustment. Our participant recruitment was conducted through Prolific, which mandates a minimal hourly reward requirement. Consequently, we have opted not to replicate the initial payments adjusted for inflation
4	Study 4 reward	participants were informed that randomly selected respondents would receive an actual reward based on their choices	all participants were paid a fixed amount for the single data collection with all the studies combined. We did not randomly select participants for reward	simple, equal and fair pay for all participants

We made several adjustments to the target article’s stimuli. In Study 1a, the original target event of Item 2 identified by Fox & Rottenstreich [1] was the University of Illinois winning the scoring title. In 2003, the Big Ten Conference in Women’s lacrosse consisted of 11 teams. However, in recent years, the league has been reduced to seven teams, with the University of Illinois no longer participating. Consequently, we adjusted this item to estimate the University of Michigan’s winning rate. Fox & Rottenstreich [1] did not explicitly specify that the Big Ten Conference had 11 teams. They assumed that some of their participants would know the information, whereas others would not, considering both 1/10 and 1/11 as ignorance priors. In contrast, given our broader sample, we needed to explicitly inform our participants that there were seven teams in the league so that they can make an informed decision. Therefore, our ignorance prior for this item was set at 1/7. This is a deviation from the target, but we felt a needed and crucial one because without this information and/or asking participants for their knowledge about that league, we are assuming too much and many things can go wrong.

Likewise, in both Item 3 of Studies 1a and 4, Fox & Rottenstreich [1] originally targeted General Motors’ (GM’s) stock price rising more than any other stock on the DJIA. However, the components of the DJIA have undergone changes over time and GM is no longer a constituent of the DJIA. Consequently, we have replaced GM with IBM, which at the time of data collection was a component of the DJIA (2024).

### 2.4. Procedure

We reconstructed the target’s survey items of Studies 1a, 1b, 3 and 4 and adjusted it to an online Qualtrics survey based on the information provided in the article.

Participants were initially presented with the informed consent form and a detailed outline of the study’s requirements and procedures. To proceed, they were asked to indicate their consent with four questions confirming their eligibility, understanding and agreement with study terms, which they must answer with a ‘yes’ and required responses in order to proceed to the study. Three of the four questions also served as attention checks, with the options order being rotated (yes, no, not sure). Failing those attention checks meant that the participants did not indicate consent and therefore could not embark on the study.

Upon confirming their consent and demonstrating understanding of the study instructions, participants were randomly assigned participants to either the case-prime condition or the class-prime condition (‘evenly presented’ randomizer in Qualtrics). Within each condition, participants answered all scenarios from the four studies of the target article in a randomized order.

We implemented two questions of competing hypotheses for all scenarios in Studies 1a, 1b and 3 (see clarification above). We note that in Study 1a of the target article, it was reported that participants encountered three pairs of complementary hypotheses, yet Fox & Rottenstreich [1] did not specify whether each pair was presented concurrently or if all six questions (3 items × 2 hypotheses) were presented in a counterbalanced manner. Given the repetition of the complementary hypotheses in all five scenarios, we were concerned that randomizing the order would confuse participants, and so presented the pairs in fixed order, where the ‘happen’ hypothesis is always presented first, and the ‘not happen’ hypothesis is always presented second.

In both conditions, participants made a series of probability prediction queries including several numerical probability estimations and a two-option decision-making task. Under the case prime, the queries were designed to facilitate a binary case partition with an ignorance prior of 1/2. Under the class prime, the queries were designed to facilitate an *n*-fold class partition with an ignorance prior of 1/*n*. At the end of the experiment, participants answered a number of funnelling and demographic questions.

### 2.5. Manipulations

#### 2.5.1. Partition priming (between-groups)

Each participant was randomly assigned to complete tasks involving likelihood estimation in either the case-prime or the class-prime condition with either case or class formulations of the same queries.

#### 2.5.2. Scenario (within-groups)

Each participant completed two types of tasks: numerical probability estimation task and two-option decision-making task. For the numerical probability estimation task, participants rated the likelihood of an event’s occurrence in percentages. In the decision-making task, the first option was consistent across both conditions, whereas the second option included an ignorance prior aligned with either case or class formulations of the same event. Participants were directed to select between the two options. Although participants’ tendency to select the second option correlated with their probability estimation of the occurrence of the target event associated with the second option, they were not required to provide numerical responses during the decision-making task.

### 2.6. Measures

#### 2.6.1. Replication

##### 2.6.1.1. Studies 1a, 1b and 3: probability estimation

Based on the information presented in the target article, the participants in Fox and Rottenstreich’s study [1] recorded probability estimations in both fractions and decimals (e.g. 1/2 and 0.5). In our study, we maintained the probability scale from 0 to 1 as used in the target article, but we specifically instructed participants to record probabilities as percentages. The percentage scale ranged from 0% to 100%, with 0% indicating that the target event will not happen and 100% indicating that the target event will happen (table 5).

##### 2.6.1.2. Study 4: Choice

The decision outcome is either option 1 or option 2 (table 6).

### 2.7. Deviations

We made a few needed adjustments to the target article’s studies and stimuli and summarize those in table 7.

### 2.8. Evaluation criteria for replication findings

We pre-registered our strategy to evaluate our conclusion of whether the target article successfully replicated overall based on the number of studies in which our findings indicated a signal in the same direction as the target article, per the following: successful: three or four out of four studies; a failed replication: no studies; mixed findings: one or two studies. After data collection when writing our results, we realized that these criteria were not sufficiently specified, and we extended the spirit of these criteria to also apply to the three items in Study 1a, such that two and three out of the three items would be considered successful. We explain this in greater detail in our analyses in §§3 and 4.

### 2.9. Replication closeness evaluation

We provide details on the classification of the replications using the criteria by LeBel *et al.* [24] in table 8. Given the adjustments to the stimuli, data analysis, our extensions and the unified design, we summarized the replication as being between direct and conceptual, a ‘close to far’ replication. Much was based on the target article, but any replication that would aim to repeat the target’s method would need to make adjustments and our extensions and planned analyses overall help strengthen the replication, improve accuracy and reduce noise.

**Table 8.** Classification of the replication, based on LeBel *et al*. [24].

design facet	replication	details of deviation and severity (minor/major)
effect/hypothesis	same	
IV construct	same	
DV construct	same	
IV operationalization	similar	we made minor adjustments to the scenarios to update them to current times (table 7)
DV operationalization	different	major: across all studies we elicited both the affirmative and the negative of the probability (will versus will not occur)
IV stimuli	same	
procedural details	different	major procedure: Fox & Rottenstreich [1] recruited different participants for Studies 1a, 1b, 3 and 4. We combined the four studies into a cohesive single data collection. Each participant completed tasks from all four studies compensation: Fox & Rottenstreich [1] employed varying compensation schemes across the four studies we chose to replicate, as detailed in table 4. We have adhered to the standard payment rates on Prolific
contextual variables	same	
population (e.g. age)	different	major: Fox & Rottenstreich’s [1] participants were exclusively undergraduate and MBA students of US universities. Our participants were drawn from the general US population
replication classification	close to far replication	

Criteria for evaluation of replications by LeBel *et al.* [24]. ‘Similar’ category was added to the LeBel *et al.* [24] typology to refer to minor deviations or extensions aimed to adjust the study to the target sample that are not expected to have major implications on replication success.

### 2.10. Outliers and exclusions

We did not classify any cases as outliers. We included all the data collected in our analysis for those who successfully completed the entire study.

## 3. Results

We summarize the descriptives of replication results in table 9 and statistical tests in tables 10 and 11. The following analyses were performed with R v. 4.4.0 [15] with support from JAMOVI v. 2.4.8 [25] and their ‘jmv’ R package.

**Table 9.** Studies 1a, 1b and 3: descriptive statistics of probability estimation of ‘happen’ target events.

study	item	case prime (*n* = 300)				class prime (*n* = 303)			
		ignorance prior	*med* (%)	*M* (%)	s.d. (%)	ignorance prior	*med* (%)	*M* (%)	s.d. (%)
1a	weather	1/2 (50%)	23.75	31.93	22.00	1/7 (14%)	15.00	26.66	20.92
	sports	1/2 (50%)	20.00	27.74	18.50	1/7 (14%)	15.00	25.06	18.41
	business	1/2 (50%)	10.00	19.77	21.61	1/30 (3%)	5.00	17.97	21.18
1b	weather	1/2 (50%)	60.00	56.83	23.39	5/7 (71%)	71.00	66.91	18.17
3	offer	1/2 (50%)	70.00	59.93	29.54	1/10 (10%)	50.00	47.53	32.56

*med*, median judged probability; *M*, mean; s.d., standard deviation; *n*, condition sample size. The units of *med*, *M* and s.d. are percentages.

**Table 10.** Studies 1a, 1b and 3: results of Mann–Whitney *U*-test and Welch’s *t*-test comparing probability estimation (‘happen’ events).

		Study 1a			Study 1b	Study 3
		Item 1	Item 2	Item 3		
Mann–Whitney *U*	*U*	40 166*	40 551*	41 854	33 026***	35 824***
	*p*	0.013	0.021	0.092	<0.001	<0.001
	rank biserial *r* and 95% CI	0.12 [0.03, 0.21]	0.11 [0.02, 0.20]	0.08 [−0.01. 0.17]	−0.27 [−0.36, −0.19]	0.21 [0.12, 0.30]
Welch’s *t*	*t*	3.02**	1.78	1.03	−5.90***	4.90***
	d.f.	598.8	600.9	600.6	563.8	596.5
	*p*	0.003	0.075	0.304	<0.001	<0.001
	Cohen’s *d* and 95% CI	0.25 [0.09, 0.41]	0.15 [−0.14, 0.31]	0.08 [−0.08, 0.24]	−0.48 [−0.64, −0.32]	0.40 [0.24, 0.56]

*p < 0.05, **p < 0.01, ***p < 0.001. We aligned all effects to go in the same direction with case condition higher than class condition, yet Study 1b was meant to demonstrate class condition higher than case condition, and so the effects are coded as negative.

**Table 11.** Studies 1a, 1b and 3: proportions of response aligned with ignorance priors (‘happen’ events).

condition	condition	Study 1a Q1	Study 1a Q2	Study 1a Q3	Study 1b	Study 3
case	case-primed responses	19.33%	11.33%	9.67%	19.67%	5.00%
	class-primed response	*33.67%*	*41.33%*	*41.00%*	*22.67%*	*15.67%*
	neither	47.00%	47.34%	49.33%	57.66%	79.33%
class	case-primed responses	10.56%	9.24%	7.59%	8.58%	8.25%
	class-primed response	*48.18%*	*50.17%*	*40.92%*	*40.92%*	*29.70%*
	neither	41.26%	40.59%	51.49%	50.50%	62.05%

Responses falling within ±5% of the ignorance prior were considered to be influenced by partition priming (aligned with ignorance priors). Case comparisons are underlined and class comparisons are italicized to aid readers.

### 3.1. Replication

#### 3.1.1. Studies 1a, 1b and 3

To mirror the target article’s analyses, we conducted a series of Mann–Whitney *U-*tests and Welch’s *t*-tests to compare the case and class conditions on probability estimations in Studies 1a, 1b and 3. Additionally, we ran chi-squared tests to compare the case and class conditions on the prevalence of responses aligning with ignorance priors.

A pre-registered deviation from the target was that we based our assessment of overall support for the hypotheses based on the estimations, rather than the comparison of the arbitrary categorization of match with ignorance priors. We felt that the theory of partition dependence was more aligned with the overall shift in preference rather than whether some participants were closer to ignorance priors, so we supplemented our reporting with chi-squared for the sake of consistency with the target and completeness.

##### 3.1.1.1. Probability estimations

**Figure 1.**
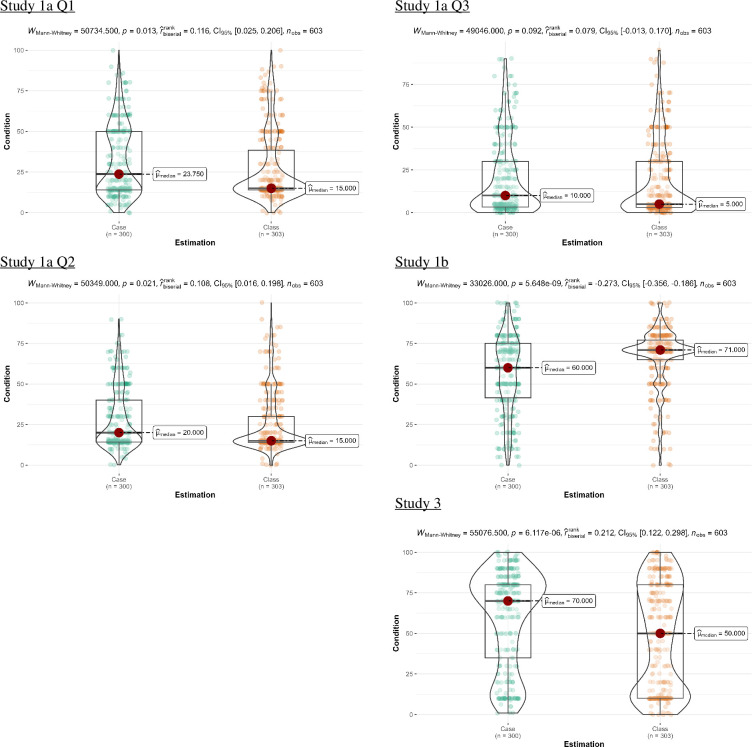
Studies 1a, 1b and 3: comparison of estimations between the case and class conditions (Mann–Whitney *U*-test). Violin plots of the distribution of responses, boxplots displaying the median, first and third quartiles, and the red circle identifying the median value.

We summarize descriptive statistics of Studies 1a, 1b and 3 in table 9. Our findings are presented in table 10 and plotted in figure 1.

Our Mann–Whitney *U*-tests revealed differing probability judgements between the two conditions in the expected direction, except for Study 1a Item 3 (business), which showed no support for differences. Our Welch’s *t*-tests found similar results, yet with no support for Study 1a Item 2 (sports). We found *higher* probability judgements in the case-prime condition compared to the class-prime condition for Study 1a Item 1 (weather) and Study 3 (offer), where the ignorance priors under the case formulation were higher than those under the class formulation. However, in Study 1b, as expected, we identified *lower* probability judgements in the case-prime condition compared to the class-prime condition, where the ignorance priors under the case formulation were lower than those under the class formulation (see table 9 and the lower panel of table 10).

##### 3.1.1.2. Ignorance priors

**Figure 2.**
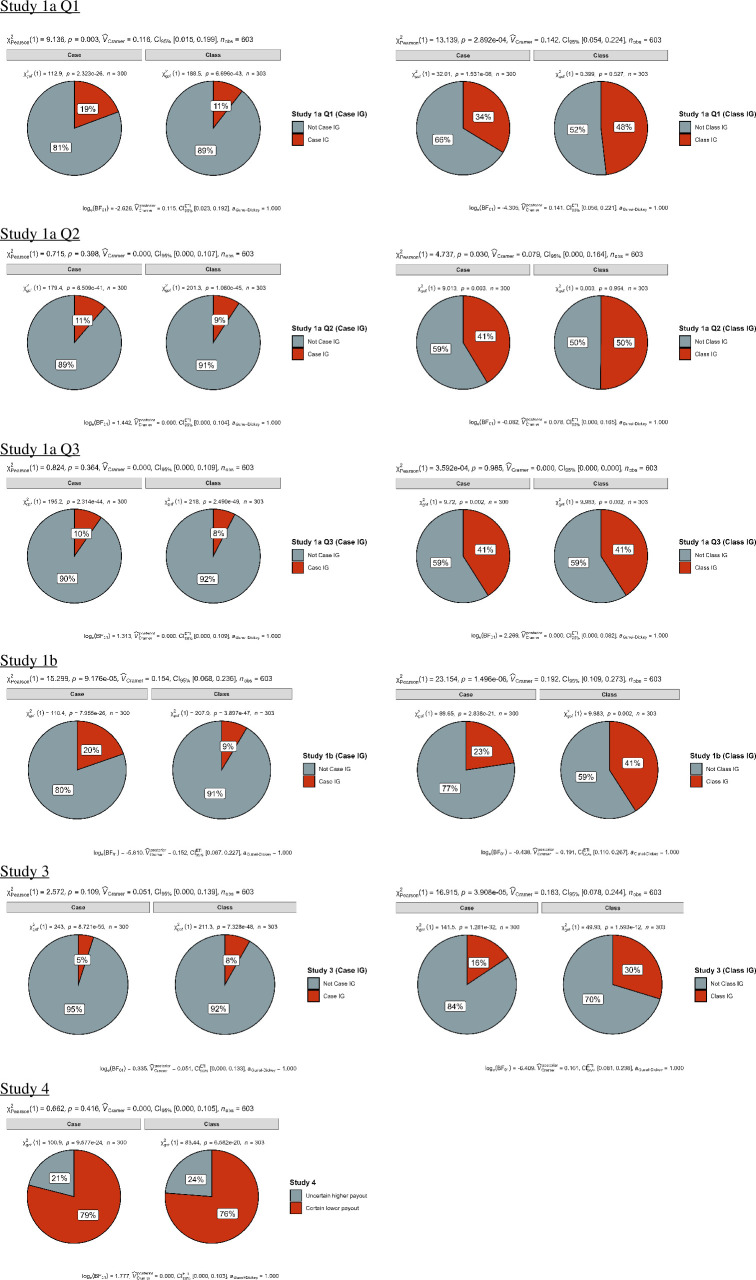
Studies 1a, 1b, 3 and 4: comparison of ignorance priors (1a/b and 3) and decisions (4) between the case and class conditions (chi-squared tests). For Studies 1a, 1b and 3, the left panel shows comparison between responses that aligned with case-primed ignorance priors and those that did not. The right panel shows comparison between responses that aligned with class-primed ignorance priors and those that did not. For Study 4, the left panel depicts the proportions of participants choosing the uncertain higher payment option versus the certain lower payment option in the case condition and the right panel shows the proportions of these choices in the class condition.

We also examined the proportions of responses aligned with ignorance priors across different conditions and items, summarized in table 11 and plotted in figure 2. Specifically, we examined for the two case and class conditions: (i) proportions of judgements aligned with the case-prime ignorance prior (1/2); and (ii) proportions of judgements aligned with the class-prime ignorance prior (1/*n* or *x*/*n*).

We pre-registered the decision to classify all responses falling within ±5% of the ignorance prior as influenced by partition priming, a criterion that was not well specified in the target article. For instance, in Study 1b, the ignorance prior of the case condition was 50%, and responses ranging from 45% to 55% were identified as being case primed. Similarly, with an ignorance prior of 71% for the class condition, responses falling within the range of 66–76% were classified as class primed. No item had overlapping response ranges. For Item 3 in Study 1a, responses ranging from 0% to 8% were classified as being class primed. In the target article, Fox & Rottenstreich [1] utilized varying definitions of response ranges for case-primed or class-primed conditions across different items. For instance, some items employed a ±1% range (e.g. 0.14 or 0.15 for Item 1 of Study 1a), while others were rounded to two decimal places (e.g. 0.03 for Item 3 of Study 1a), and certain items utilized exact numbers (e.g. 0.1 for Study 3).

We performed a series of chi-squared tests comparing the proportions of case-primed and class-primed responses in the two experimental conditions (table 12; figure 2). Our results demonstrated the varying influence of priming conditions on responses’ alignment with ignorance priors across the different scenarios.

**Table 12.** Studies 1a, 1b and 3: *χ*^2^‐test comparing the proportions of case-primed and class-primed responses in the two experimental conditions (‘happen’ events).

	study	case-primed response	class-primed response
*χ*^2^ (d.f. = 1)	1a Q1	9.14**	13.14***
	1a Q2	0.72	4.74*
	1a Q3	0.82	0.00
	1b	15.30***	23.15***
	3	2.57	16.92***
*p*	1a Q1	0.003	<0.001
	1a Q2	0.398	0.030
	1a Q3	0.364	0.985
	1b	<0.001	<0.001
	3	0.109	<0.001

*N*_case_ = 600, *N*_class_ = 603. *p < 0.05, **p < 0.01, ***p < 0.001.

For Study 1a Q1 (weather) and 1b (weather), we found support for greater proportions of both case-primed and class-primed responses in the respective conditions, with higher proportion of case-primed responses in the case condition and a greater proportion of class-primed responses in the class condition. For Study 1a Q2 (sports) and Study 3 (offer), we only found support for the class-primed responses, as participants in the class condition made these responses more frequently than those in the case condition. For Study 1a Q3 (business), we found no evidence for alignment with either case-primed or class-primed responses.

### 3.1.2. Study 4

We conducted a two-proportion *z*-test and found no support for differences in the proportion of participants selecting the second option (uncertain high payment) between the case and the class condition, with proportions of 21.00% (case) and 23.76% (class), *z* = 0.81, *p* = 0.415, Cohen’s *h* = 0.08, 95% CI [−0.11, 0.27] (see bottom of figure 2).

### 3.2. Replication evaluation

To assess the reproducibility of our results compared to those of the target article, we employed the paradigm outlined by LeBel *et al.* [26], examining the existence of a signal and comparing confidence intervals with the effect size reported in the target article. We summarize the outcomes of this comparison in table 13.

**Table 13.** Replication: comparison of effects between the target article and our replication using the LeBel *et al.* [26] replication evaluation criteria.

study		target article	replication	
estimations		Cohen’s *h* and CI	Cohen’s *h* and CI	interpretation
1a	Item 1	0.65 [0.24, 1.06]	0.30 [0.14, 0.46]	signal—inconsistent, smaller
	Item 2	0.65 [0.24, 1.06]	0.18 [0.02, 0.34]	signal—inconsistent, smaller
	Item 3	0.65 [0.24, 1.06]	0.00 [−0.16, 0.16]	no signal—inconsistent
1b		0.67 [0.07, 1.28]	0.43 [0.25, 0.60]	signal—inconsistent, smaller
3		0.62 [0.16, 1.08]	0.41 [0.21, 0.60]	signal—inconsistent, smaller
4		0.32 [0.00, 0.65]	0.08 [−0.11, 0.27]	no signal—inconsistent
ignorance priors		Cohen’s *d* and CI	Cohen’s *d* and CI	interpretation
1a	Item 1	0.55 [0.13, 0.96]	0.25 [0.09, 0.41]	signal—inconsistent, smaller
	Item 2	0.55 [0.13, 0.96]	0.15 [−0.14, 0.31]	signal—inconsistent, smaller
	Item 3	0.55 [0.13, 0.96]	0.08 [−0.08, 0.24]	no signal—inconsistent
1b		−0.87 [−1.51, −0.22]	−0.48 [−0.64, −0.32]	signal—inconsistent, smaller
3		0.47 [0.00, 0.93]	0.40 [0.24, 0.56]	signal—consistent

DVs: Studies 1a/b and 3 are probability estimations, and Study 4 is decision outcome. CI = 95% confidence intervals. Upper panel presents Cohen’s *h* comparing proportions of class-primed responses in the two experimental conditions (Studies 1a, 1b and 3, ‘happen’ events) and proportions of choice option (Study 4). Lower panel presents Cohen’s *d* comparing probability judgements (Studies 1a, 1b and 3). We aligned all effects of Welch’s *t*-tests (Cohen’s *d*) to go in the same direction with case condition higher than class condition, yet Study 1b was meant to demonstrate class condition higher than case condition, and so the effects are coded as negative. In the target article, we derived Cohen’s *h* and Cohen’s *d* in Study 1a by combining responses across all three items, as statistics for individual items were unavailable. For Study 1b in the target article, Cohen’s *d* was derived from the *p*-value reported in the target article. We used the LeBel *et al.* [26] replication evaluation criteria, summarized in the electronic supplementary material, S4a/b. Briefly: ‘signal—consistent’: the CI for the replication effect size (ES) excludes zero and includes the original study’s ES; ‘signal—inconsistent, smaller’: the replication ES CI excludes zero and the upper CI is smaller than the original study’s ES; ‘no signal—inconsistent’: the replication ES CI includes zero and does not overlap with the original study’s ES.

After the analysis, we realized that our pre-registered criterion for concluding effects for Study 1a given the three items was under-specified, given that there are three items, of which two were supported, and we did not indicate in advance how we would treat such a case. Post-analysis, in the spirit of our pre-registered criterion that if the majority of studies are supported, then we conclude a successful replication, we applied similar criteria to the items in Study 1a, and we note that as a deviation/expansion. We decided to conclude support for an effect if two out of three items were supported, resulting in our classification of Study 1a as a successful replication. We also were not clear enough which of all the tests used, we would use as the core analysis for the evaluation of replication, and decided to focus on the main Mann–Whitney *U*-test as the most closely aligned with our pre-registration.

Given that three of the four studies were supported for the core main dependent variable, based on our pre-registered criteria we concluded our attempt as a (mostly) successful replication.

### 3.3. Extension: a three-way mixed ANOVA for Studies 1a (cases 1/2/3), 1b and 3

One of the advantages of our unified design combining several items and studies is the ability to run a more comprehensive analysis comparing the effects across the different scenarios. We also added an extension going beyond the replication of asking for the probability of something to ‘happen’ and also asked about the probability of something to ‘not happen’. We generally expected no differences between ‘happen’ and the reverse of ‘not happen’, and had no specific predictions, and therefore classified this analysis as exploratory. This approach served to examine differences across scenarios and in competing hypotheses framing, as a potential examining of the generalizability of the phenomenon.

**Figure 3.**
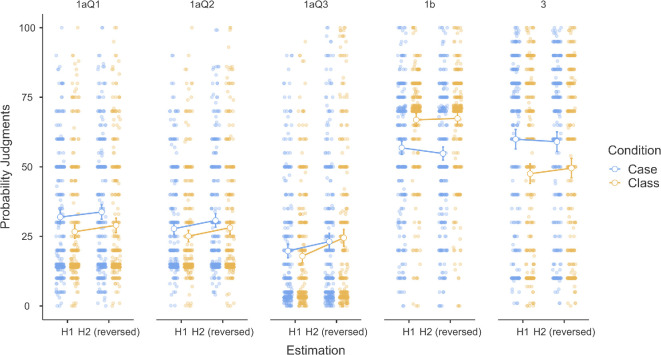
Three-way mixed ANOVA of condition, scenario and evaluation type on probability judgements. ‘Happen’ refers to participants’ evaluations of the probability that a target event will occur. ‘Reverse not happen’ refers to the reverse probability estimation calculated as 100% minus the probability of the same event not happening. Plotted using JAMOVI [25].

We conducted a three-way mixed ANOVA to examine the impact of condition (2 between conditions; case versus class), question (5 within conditions; Study 1a Item 1, Study 1a Item 2, Study 1a Item 3, Study 1b and Study 3) and evaluation type (2 within conditions; happen versus reverse not happen) on participants’ probability judgements.^1^ We plot the results in figure 3.

We found support for a main effect of question (*F*(4,2404) = 446.96, *p <* 0.001), and an interaction between question and condition (*F*(4,2404) = 24.32, *p* < 0.001), which are both to be expected given that Study 2 has the deliberate reversal effect. We also found support for a main effect of evaluation type (*F*(1,601) = 19.32, *p* < 0.001), with a weak interaction between evaluation type and condition (*F*(1,601) = 4.17, *p* = 0.042). We found no support for the main effect of condition on probability judgements (*F*(1,601) = 2.05, *p* = 00.153) or for a three-way interaction between evaluation type, question and condition (*F*(4,2404) = 1.42, *p* = 0.227).

To explore whether participants’ estimations of two competing hypotheses summed to 100% and whether this interacted with condition and scenario, we focused on *post hoc* tests of specific contrasts of interest.^2^ We found support for differences between estimations of two complementary events in Study 1a Q3 in the class condition (*t* = −16.24, *p* < 0.001). Specifically, participants’ average assessment of ‘happen’ probabilities (*M* = 17.97, s.e. = 1.23) was lower for ‘happen’ than for reverse ‘not happen’ (*M* = 24.46, s.e. = 1.54). For all other pairs, there was no evidence of a difference. In other words, only in Study 1a Q3 under the class condition did the sum of the two events fail to equal 100%. All other comparisons showed no differences, suggesting that participants’ estimations for two mutually exclusive events generally summed to 100%.

### 3.4. Extension: negative complementary events (Studies 1a, 1b and 3 exploratory analysis)

We examined whether participants’ likelihood judgements of negative complementary events displayed a consistent or divergent pattern of reliance towards partition-dependent priors across Studies 1a, 1b and 3. The Mann–Whitney *U*-tests indicated differing probability judgements between the case-prime and class-prime conditions for Items 1 and 2 of Study 1a, Study 1b and Study 3, while no difference was found for Item 3 of Study 1a (table 15). Welch’s *t*-tests found support for lower probability judgements in the case condition for Item 1 of Studies 1a and 3, where ignorance priors under case prime were lower than that under class prime, and higher probability judgements for Study 1b in the case condition where ignorance priors under case prime were higher than that under class prime (tables 14 and 15). Welch’s *t*‐test failed to find support for an effect of condition on Items 2 and 3 of Study 1a.

**Table 14.** Studies 1a, 1b and 3: descriptive statistics of probability estimation of ‘not happen’ target events.

study	item	case prime (*n* = 300)				class prime (*n* = 303)			
		ignorance prior	*med* (%)	*M* (%)	s.d. (%)	ignorance prior	*med* (%)	*M* (%)	s.d. (%)
1a Q1	weather	1/2 (50%)	72.00	66.23	23.12	6/7 (86%)	85.00	71.04	22.42
1a Q2	sports	1/2 (50%)	80.00	69.28	21.01	6/7 (86%)	85.00	71.89	21.57
1a Q3	business	1/2 (50%)	90.00	76.89	25.36	29/30 (97%)	90.00	75.54	28.08
1b	weather	1/2 (50%)	45.50	45.20	23.48	2/7 (29%)	29.00	32.58	18.20
3	offer	1/2 (50%)	30.00	40.95	29.82	9/10 (90%)	50.00	50.47	32.19

*med*, median judged probability; *M*, mean; s.d., standard deviation; *n*, condition sample size. The units of *med*, *M* and s.d. are percentages.

**Table 15.** Studies 1a, 1b and 3: Mann–Whitney *U*-test and Welch’s *t*-test comparing probability estimation of ‘not happen’ (complementary hypotheses) target events.

		Study 1a			Study 1b	Study 3
		Item 1	Item 2	Item 3		
Mann–Whitney *U-*test	*U*	40 568*	41 078*	44 869	30 615***	38 062***
	*p*	0.022	0.400	0.786	<0.001	<0.001
	rank biserial *r* and 95% CI	0.11 [0.02, 0.20]	0.10 [0.00, 0.19]	0.01 [−0.08. 0.11]	−0.32 [−0.41, −0.24]	0.16 [0.07, 0.25]
Welch’s *t*	*t*	2.59*	1.50	−0.62	−7.37***	3.77***
	d.f.	600.0	600.8	596.0	563.2	598.4
	*p*	0.010	0.133	0.536	<0.001	<0.001
	Cohen’s *d* and 95% CI	0.21 [0.05, 0.37]	0.12 [−0.03, 0.28]	−0.05 [−0.21, 0.11]	−0.60 [−0.76, −0.44]	0.31 [0.15, 0.47]

Probability estimations were recorded in percentages. *p < 0.05, **p < 0.01, ***p < 0.001. For the ‘happen’ events, effect sizes were recorded in their original scales (table 10). For the ‘not happen’ events presented in this table, effect sizes were recorded in reverse (negative values were converted to positive, and positive values converted to negative) to aid direct comparison. For example, consider the scenario of Study 3: for the ‘happen’ event, the case ignorance prior was 1/2 and the class ignorance prior was 1/10. For the ‘not happen’ event, the case ignorance prior was 1/2 and the class ignorance prior was 9/10. The effect sizes for ‘not happen’ events were recorded in reverse to facilitate comparison. By aligning the direction of the effect sizes, it is easier to assess whether the patterns of partition dependence are consistent across different scenarios (happen versus not happen).

### 3.5. Extension: order effect (Study 1a Item 3 and Study 4)

In the original study, the rationale for Study 4 was to investigate whether the reliance on ignorance priors depended on using a numerical response scale. In Item 3 of Study 1, participants assessed the numerical probability of a stock price rising to the highest level in the DJIA. In contrast, Study 4 required participants to evaluate the probability and make a decision without providing numerical responses. Fox & Rottenstreich [1] conducted Studies 1a and 4 independently with different participants. We conducted all four studies with the same participants, and given the similarity between the two studies, we explored order effects for the display of Study 1a Item 3 and Study 4, using a binomial logistic regression analysis.

We initially pre-registered to test this order effect only for Study 4, following the rationale of the target article, which tested whether reliance on ignorance priors was tied to explicit numerical probability after significant results from previous studies including Study 1a. However, in our analysis, we felt it would also be valuable to test this order effect on Study 1a Item 3. We document this change in the ‘Pre-registration plan versus final report’ table in the electronic supplementary material. Therefore, we added a 2 ×2 factorial ANOVA on Study 1a Item 3, examining the effects of condition (case versus class) and order (judgement first versus decision first) on participants’ probability estimations.

**Figure 4.**
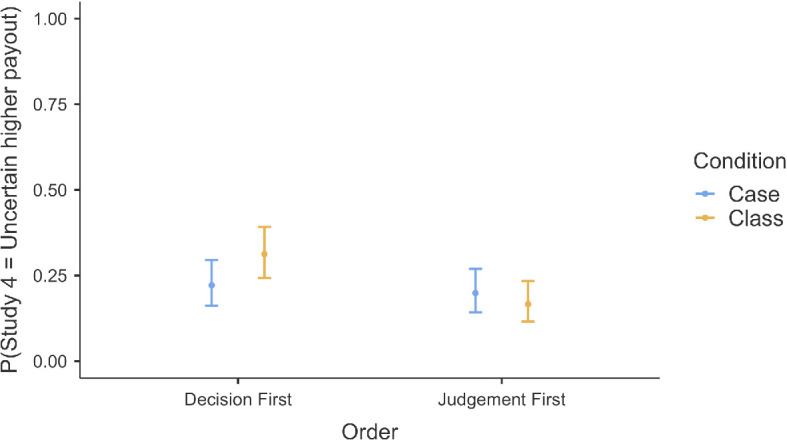
Order effect analysis: probability of choosing the uncertain higher payout in Study 4 based on task order (Study 1a Item 3 versus Study 4) and condition.

We conducted a binomial logistic regression to examine the effects of order (Study 1a Item 3 judgement first versus Study 4 decision first) and condition (class versus case) on the likelihood of participants choosing between two financial options: option 1 (receive $10 for sure) and option 2 (receive $50 if the stock whose price per share rises the most on the DJIA today is IBM). Our results showed no support for the effect of order (*b* = − 0.14, s.e. = 0.28, *z* = − 0.48, *p* = 0.628), condition (*b* = 0.47, s.e. = 0.27, *z* = 1.77, *p* = 0.077) or an interaction between order and condition (*b* = − 0.69, s.e. = 0.40, *z* = −1.72, *p* = 0.085) on the likelihood of choosing option 2 in Study 4 (figure 4).

We additionally conducted a two-way ANOVA to examine the effects of order (Study 1a Item 3 judgement first versus Study 4 decision first) and condition (case versus class) on participants’ probability estimations for Study 1a Item 3. We found no support for a main effect of order (*F*(1,599) = 0.17, *p* = 0.684), a main effect of condition (*F*(1,599) = 1.03, *p* = 0.310) or an interaction between order and condition (*F*(1,599) = 0.67, *p* = 0.412).

In summary, neither the order of task presentation nor the condition, nor their interaction, had an impact on participants’ choices in Study 4 and probability estimations in Study 1a Item 3.

### 3.6. Order effects for the unsupported study (Study 1a Item 3)

We pre-registered that if we failed to support our hypotheses in one or more studies, we would run additional analyses taking into account the presentation order of the unsupported studies with a stricter alpha of 0.005. Since there was no evidence for the hypotheses with question 3 of Study 1a (1aQ3) from any statistical test we conducted (tables 10 and 12), we proceeded with additional analyses. Specifically, we ran two sets of Mann–Whitney *U*-test and Welch’s *t*-test to compare the probability estimations between the case and class conditions. One set included participants who completed the 1aQ3 task first, and the other included those who did not complete the 1aQ3 task first.

For participants who completed Study 1a Item 3 first, the results of Welch’s *t*‐test did not meet the stricter alpha level of 0.005, *t* = 2.01, *p* = 0.047, Cohen’s *d* = 0.38, 95% CI [0.00, 0.75]. However, Mann–Whitney *U*-test indicated a difference in the distribution of probability estimation between the case (*M* = 21.92, s.d. = 21.42) and the class condition (*M* = 13.94, s.d. = 20.63), *U* = 1068, *p* = 0.004.

For participants who completed other tasks first, our results failed to find support for a difference between the two conditions, *t* = 0.20, *p* = 0.842, Cohen’s *d* = 0.02, 95% CI [−0.16, 0.19], which overlapped with the CI of those completed 1aQ3 first. The Mann-Whitney *U* test returned *U* = 29 325, *p* = 0.606.

In summary, the results indicated differences in probability estimations between the case and class conditions for participants who completed the 1aQ3 task first, as supported by the Mann–Whitney *U* test. However, this difference was not supported by Welch’s *t*‐test under an alpha level of 0.005. The effect of condition on probability assessments was not observed in participants who completed other tasks (other than 1aQ3) first, as both Mann–Whitney *U* and Welch’s *t*-tests failed to find support for a difference between conditions. The confidence intervals of Cohen’s *d* for those who did not complete 1aQ3 first overlapped with those who did. Based on the overall evidence, we concluded that the impact of order on the observed effect was likely to be very small.

## 4. Discussion

We conducted a replication and extension Registered Report of Fox & Rottenstreich [1] to revisit their findings on partition-dependent ignorance priors in probability judgements and decisions. We summarize the comparison of effects between our findings and the target article’s using the LeBel *et al.* [26] criteria in table 13. Overall, we concluded our replication as (mostly) successful with effects generally weaker than the ones reported in the target article.

Specifically, we successfully replicated Studies 1a Items 1 and 2 (2 out of 3 items), 1b and 3, and failed to find support for Study 1a Item 3 and for Study 4. Effect sizes were only consistent with those reported in the target article for Study 3, with all other studies having weaker effects than the target’s in the same direction. These mostly successful yet mixed results suggest that the phenomenon might be more nuanced than expected, with some aspects robust and holding as is over time, yet with others requiring better understanding to be applicable across different contexts and time.

### 4.1. Replication

Study 1a examined partition dependence under near ignorance when participants had limited information. For Item 1 (weather), participants exhibited a reliance on partition-dependent ignorance priors. Mann–Whitney *U* tests and Welch’s *t*-tests showed that probability assessments were higher in the case-prime condition with an ignorance prior of 1/2 than in the class-prime condition with an ignorance prior of 1/*n* (1/*n* < 1/2). Chi-squared tests revealed that there were more responses aligned with ignorance priors under the case formulation (1/2 ± 5%) in the case condition than in the class condition. Similarly, there were more responses aligned with ignorance priors under the class formulation (1/*n* ± 5%) in the class condition than in the case condition.

We found support for Item 2 (sports competition) when using Mann–Whitney *U* tests and chi-squared tests comparing class-primed judgements in the two conditions, yet not when applying Welch’s *t*-tests and chi-squared tests comparing case-primed judgements. We did not observe support for partition dependence in Item 3 (business) using any statistical tests, also when examining order effects.

Study 1b focused on probability judgements under near ignorance when the class prime was expected to facilitate a higher default probability (5/7) than the case prime (1/2). Participants’ judgements showed a bias towards partition-dependent ignorance priors. Mann–Whitney *U* tests and Welch’s *t*-tests indeed indicated lower probability judgements in the case-prime condition compared to the class-prime condition. Chi-squared tests confirmed that class-primed responses (5/7 ± 5%) were more frequent in the class condition than in the case condition. Similarly, case-primed responses (1/2 ± 5%) were more frequent in the case condition than in the class condition.

Study 3 examined partition-dependent probabilities under uncertainty, where participants could apply relevant knowledge to some degree. Participants were asked to make probability judgements similar to those in Studies 1a and 1b. Mann–Whitney *U* tests and Welch’s *t*-tests showed higher probability judgements in the case-prime condition (1/2) than in the class-prime condition (1/10). Chi-squared tests indicated more class-primed responses (1/10 ± 5%) in the class condition than in the case condition, However, we found no support for differences between the proportions of case-primed responses (1/2 ± 5%) in the two conditions.

Study 4 investigated decision making under ignorance, where participants made categorical decisions without providing numerical responses. A two-proportion *z*-test suggested no difference in participants’ likelihood of choosing the high reward option with higher risk (option 2). Our replication results did not provide evidence for partition dependence in decision making.

It is also worth noting that the target article only compared class-primed responses and did not provide data on proportions of case-primed responses nor conduct related tests apart from Study 1a (table 2). In our chi-squared tests, we tested both and found more class-primed responses in the class condition than in the case condition for four items, with more case-primed responses observed in the case condition for only two items (table 16). One possible explanation for this pattern could be that class-primed responses are better aligned with standard probability theories.

**Table 16.** Studies 1a (Q1–Q3), 1b and 3: summary of support for core hypothesis using the different tests.

sudy	Mann–Whitney *U*	Welch’s *t*	*χ²* (case primed)	*χ²* (class primed)
1a Q1 (weather)	supported*	supported**	supported**	supported***
1a Q2 (sports)	supported*	not supported	not supported	supported*
1a Q3 (business)	not supported	not supported	not supported	not supported
1b (weather)	supported***	supported***	supported***	supported***
3 (job offer)	supported***	supported***	not supported	supported***

*p < 0.05, **p < 0.01, ***p < 0.001. Replication success evaluation criteria focused on the Mann–Whitney *U*-tests.

Previous literature has shown that partition dependence could vary depending on the context and the nature of the decision-making tasks [3,5]. Theoretically, our replication findings may contribute to the literature on judgement and decision making by highlighting the context-dependent nature of partition priming. Our results suggested that individuals’ probability evaluations might be more influenced by partitioning in some contexts (e.g. everyday scenarios including Items 1 and 2 of Study 1a, Study 1b and Study 3) than in others (e.g. financial judgements such as Item 3 of Study 1a and Study 4). Practically, these results underscore the need for more testing and replications, and the need for humility and caution when applying partition-dependent probability models in different settings. For instance, it is possible that financial decision making may require better fine-tuning or altogether does not align as well with these models, suggesting that alternative approaches might be necessary for accurately predicting such decisions.

### 4.2. Extensions

We conducted several exploratory extensions without pre-registered hypotheses to examine the robustness and generalizability of partition dependence under different conditions and scenarios. Specifically, we investigated participants’ probability estimations of pairs of complementary events with a three-way mixed ANOVA contrasting: (i) the target event will happen; and (ii) the target event will not happen (reversed). We also investigated whether the sequential order of task presentation between Study 1a Item 3 and Study 4 affected participants’ judgements in Study 1a Q3 and decisions in Study 4, aiming to determine if partition dependence is tied to individuals’ explicit awareness of numerical scales.

For the core hypotheses on partition dependence, the same pattern appeared for the ‘not happen’ items, where evidence supportive of partition dependence to varying extents was observed for all items in the three studies investigating probability judgements, except for Study 1a Q3 (business), where no evidence of partition dependence was found.

For the complementary events analysis, we found differences between ‘happen’ and reverse ‘not happen’ only for Study 1a Q3 in the class condition, suggesting that participants’ probability judgements in this specific scenario did not sum to 100%. This was also the one estimation item that did not replicate well. This points to potential variations with binary additivity in subjective probability judgements. It is not immediately clear to us what makes this item special, and how much that issue impacted the replicability of the findings, and future studies may try and explore that further.

In the target article, the rationale for Study 4 was to investigate whether the reliance on ignorance priors still exhibit without using a numerical response scale. In Item 3 of Study 1, participants estimated the probability of a stock price rising to the highest level in the DJIA numerically. In contrast, Study 4 required decision making without numerical responses. Our analyses revealed no clear indication for the impact of condition, order or their interaction on the findings of either study.

### 4.3. Implications, limitations and directions for future research

Our results mostly aligned with previous research indicating that individuals’ probability judgements may exhibit biases towards partition dependence under conditions of both ignorance and uncertainty [3,4]. However, the deviation observed in some items (Study 1a Q3 and Study 4) highlights the necessity for deeper understanding of how different contexts interact with biases in probability assessment.

In this replication, we only conducted a replication of a limited number of scenarios of the target article, yet given our results we feel that this calls for a more systematic examination of more scenarios from the broader follow-up literature on this phenomenon.

One limitation of any replication study is the need to follow the methodological framework of the original studies. We aimed to closely follow Fox & Rottenstreich’s [1] analysis, yet felt that the replication would be better served by making adjustments and adding analyses and extensions to address issues that were not covered by the target article. This is a classic dilemma for replicators, weighing the pros of cons, taking into account that it is possible and even likely that by making these changes we will impact findings and will arrive at a more complex view of the phenomenon. We also conducted a comprehensive test of several studies in the target article, combining those into a single unified data collection. Our results show why such a method is so important. If replication studies were to only focus on Study 1a Item 3 or Study 4 and then fail to replicate those, they may have concluded no support for the phenomenon, possibly resulting in an misaligned conclusion or a back and forth in the community between replicators and those who have successfully observed the phenomenon, to overinvest in focusing only on those studies thereby missing the broader mostly successful framework. This also shows why making replications mainstream is so important, no single article or study can convincingly inform for or against a phenomenon, for us to establish the robustness of a phenomenon or to claim its falsification, we need several rigorous well-designed well-powered independent replications across many contexts and samples.

In summary, we see many promising directions for future research. First, expanding the range of scenarios to test the robustness of partition dependence across different contexts, particularly those involving financial judgements and rewards versus everyday scenarios. This approach would help determine whether the observed differences in the current study are consistent across a broader range of situations. Additionally, further investigations are needed to understand the cognitive processes underlying the unique findings regarding estimations of mutually exclusive events in Study 1a Q3, class condition. Future research may explore whether specific biases or heuristics are more prevalent in financial decision making and how they interact with partition dependence and influence probability judgements. Continued efforts to replicate and extend these findings with larger, more diverse samples will help establish the reliability and generalizability of ignorance priors and partition dependence. Finally, future replication studies should carefully assess the methodologies of the original studies and make appropriate adjustments while reporting necessary statistics for a clear unambiguous replication evaluation criteria. This would ensure that the replication process addresses any methodological shortcomings of the original and provides a comprehensive assessment of the robustness of the findings.

## 5. Conclusion

We conducted a Registered Report of a replication and extensions of the findings by Fox & Rottenstreich [1] on partition dependence in judgement and decision making under uncertainty and ignorance. We were mostly successful in observing partition dependence, in scenarios involving the context of weather, sports and job application, yet with weaker effects than in the target article, and with failure to find support for the effect in items involving financial contexts like stock price predictions.

## Data Availability

We provide all materials, data and code on OSF [27]. Electronic supplementary material is available online [28].

## Footnotes

^1^
We note a slight deviation from our pre-registered plan. Instead of using the original ‘not happen’ probabilities, we created a new variable called ‘reverse not happen’, calculated as Probability (reverse not happen) = 100% − Probability (not happen). ‘Happen’ and ‘not happen’ were a pair of complementary events (table 5), and in principle, their likelihood of occurrence should add up to 100%. However, participants’ assessments might have been biased, and the sum of their estimations of the two mutually exclusive events might exhibit a deviation from 100%. We felt that directly comparing participants’ assessments of ‘not happen’ and ‘happen’ probabilities without this adjustment would make it unclear whether any observed differences indicate a deviation from the sum of 100%. With no adjustment it would be difficult to interpret whether the differences were due to actual biases in estimation (e.g. 30% for a ‘happen’ event and 60% for a ‘not happen’ event) or simply because of the inherent issue that the probabilities are different (e.g. 30% for a ‘happen’ event and 70% for a ‘not happen’ event). By using this new variable, we aimed to provide a clearer comparison of how participants estimated probabilities for a pair of complementary events. This transformation ensures that the comparison between the probabilities for ‘happen’ and ‘reverse not happen’ is meaningful and directly interpretable.

^2^
We did not conduct *post hoc* tests for all interactions which our results showed evidence for. For example, although we found support for an interaction between question and condition, a direct comparison between Study 3 case and Study 3 class would not be informative because the probability estimations contained both happen and reverse not happen. Therefore, we primarily examined Tukey’s *post hoc* tests following the three-way interaction, comparing pairs of complementary events within each specific combination of question and condition (e.g. Study 1a case happen versus Study 1a case reverse not happen, Study 1a class happen versus Study 1a class reverse not happen).
